# Development of a luciferase-based reporter of transcriptional gene silencing that enables bidirectional mutant screening in *Arabidopsis thaliana*

**DOI:** 10.1186/1758-907X-3-6

**Published:** 2012-06-07

**Authors:** So Youn Won, Shengben Li, Binglian Zheng, Yuanyuan Zhao, Dongming Li, Xin Zhao, Huilan Yi, Lei Gao, Thanh Theresa Dinh, Xuemei Chen

**Affiliations:** 1 Department of Botany and Plant Sciences, Institute of Integrative Genome Biology, University of California, Riverside, CA, 92521, USA; 2 School of Life Sciences, Lanzhou University, Lanzhou, 730000, Gansu, China; 3 Laboratory of Plant Stress Ecophysiology and Biotechnology, Cold and Arid Regions Environmental and Engineering Research Institute, Chinese Academy of Sciences, 320 Donggang West Road, Lanzhou, 730000, Gansu, China; 4 School of Life Science, Shanxi University, 92 Wucheng Road, Taiyuan, 030006, China; 5 NSF ChemGen IGERT program, University of California, Riverside, CA, 92521, USA; 6 Howard Hughes Medical Institute, University of California, Riverside, CA, 92521, USA; 7 Current address: State Key Laboratory of Genetic Engineering and Institute of Plant Biology, School of Life Sciences, Fudan University, Shanghai, 200433, China

**Keywords:** Cytosine methylation, Demethylation, RdDM, MOM1, Transcriptional gene silencing, siRNA

## Abstract

**Background:**

Cytosine methylation is an important chromatin modification that maintains genome integrity and regulates gene expression through transcriptional gene silencing. Major players in *de novo* methylation guided by siRNAs (known as RNA-directed DNA methylation, or RdDM), maintenance methylation, and active demethylation have been identified in *Arabidopsis*. However, active demethylation only occurs at a subset of RdDM loci, raising the question of how the homeostasis of DNA methylation is achieved at most RdDM loci. To identify factors that regulate the levels of cytosine methylation, we aimed to establish a transgenic reporter system that allows for forward genetic screens in *Arabidopsis*.

**Results:**

We introduced a dual *35 S* promoter (*d35S*) driven *luciferase* reporter, *LUCH*, into *Arabidopsis* and isolated a line with a moderate level of luciferase activity. *LUCH* produced transgene-specific 24 nucleotide siRNAs and its *d35S* contained methylated cytosine in CG, CHG and CHH contexts. Treatment of the transgenic line with an inhibitor of cytosine methylation de-repressed luciferase activity. Mutations in several components of the RdDM pathway but not the maintenance methylation genes resulted in reduced *d35S* methylation, especially CHH methylation, and de-repression of luciferase activity. A mutation in *MOM1*, which is known to cooperate with RdDM to silence transposons, reduced *d35S* DNA methylation and de-repressed *LUCH* expression. A mutation in ROS1, a cytosine demethylation enzyme, increased *d35S* methylation and reduced *LUCH* expression.

**Conclusion:**

We developed a luciferase-based reporter, *LUCH*, which reports both DNA methylation directed by small RNAs and active demethylation by ROS1 in *Arabidopsis*. The moderate basal level of *LUCH* expression allows for bi-directional genetic screens that dissect the mechanisms of DNA methylation as well as demethylation.

## Background

Cytosine methylation is a major epigenetic mechanism that establishes transcriptional gene silencing (TGS) to maintain genome integrity and regulate gene expression in plants and mammals (reviewed in [1]). Well-known biological phenomena involving DNA methylation as an underlying mechanism include imprinting, paramutation and X chromosome inactivation. In plants, transposons and repetitive elements are methylated, thereby keeping transposons silenced and immobilized and consequently protecting the genome from damage by these mobile elements. Also, when transposons or repeats are located in the regulatory regions of genes, DNA methylation at the transposons or repeats may influence the transcription of the nearby genes through TGS.

The enzymes that initiate, maintain, and erase DNA methylation in *Arabidopsis* have been identified and characterized (reviewed in [1]). *De novo* DNA methylation, also known as RNA-directed DNA methylation (RdDM), requires DOMAIN REARRANGED METHYLTRANSFERASE2 (DRM2), which is guided to specific genomic loci by 24 nucleotide small interfering RNAs (siRNAs). siRNAs are synthesized from repeats and transposons in an RNA polymerase IV (Pol IV)-, RNA DEPENDENT RNA POLYMERASE2 (RDR2)-, and DICERLIKE3 (DCL3)-dependent manner. Pol IV is thought to transcribe these loci into single-stranded RNAs, which are then rendered double-stranded by RDR2. DCL3 dices the double-stranded RNAs into 24 nucleotide siRNAs, which are loaded into the ARGONAUTE4 (AGO4)-clade of AGO proteins (reviewed in [2]). Base-pairing between the AGO4-loaded siRNAs and nascent transcripts produced by Pol V is thought to recruit AGO4/siRNAs and DRM2 to the RdDM targets, resulting in *de novo* methylation in a sequence-specific manner (reviewed in [2]). After the initial establishment of DNA methylation, hemimethylated cytosines in CG and CHG contexts resulting from DNA replication are fully methylated by METHYLTRANSFERASE1 (MET1) and CHROMOMETHYLTRANSFERASE3 (CMT3), respectively (reviewed in [1]). The positive feedback loop in which DNA methylation promotes siRNA biogenesis, which guides *de novo* DNA methylation, needs to be kept in check to prevent the expansion of heterochromatin and the sporadic silencing of genic regions. One such mechanism is DNA demethylation. Four DNA glycosylase/lyase enzymes remove methyl cytosine through a base excision repair mechanism (reviewed in [3]). DEMETER establishes imprinting during female gametogenesis and REPRESSOR OF SILENCING1 (ROS1), DEMETER-LIKE2 (DML2) and DML3 prevent hypermethylation in vegetative tissues.

Although the enzymes that deposit or erase DNA methylation are known, how these enzymes are regulated to achieve the proper homeostasis of DNA methylation is still nebulous. Although demethylation can keep DNA methylation in check, whole genome bisulfite sequencing in the *ros1 dml2 dml3* triple mutant revealed that only a few hundred loci are hypermethylated [4] and are thus targets of demethylation. Since thousands of loci harbor DNA methylation, generate siRNAs and are targets of RdDM, it remains to be determined how most RdDM loci achieve homeostasis of DNA methylation. It is likely that other, as yet unknown, mechanisms prevent the hypermethylation of RdDM loci.

In addition to the RdDM pathway, *MORPHEUS’ MOLECULE1* (*MOM1*) impacts TGS in a complex manner usually without affecting the levels of cytosine methylation at target loci [5-7]. It encodes a protein with similarities to chromatin remodeling ATPases and silences endogenous loci and transgenes by an unknown mechanism [7]. *MOM1* exhibits a complex relationship with RdDM depending on the target loci [5]. It functions either in the same pathway as RdDM or in a parallel pathway, or it could even antagonize the silencing by RdDM. Some loci are transcriptionally suppressed by *MOM1* independently of RdDM.

Forward genetic screens in *Arabidopsis* can help reveal mechanisms that regulate DNA methylation. In fact, most of the currently known genes involved in DNA methylation or demethylation were uncovered through genetic screens. However, most prior genetic screens were based on the isolation of mutations that release RdDM to result in de-repressed reporter gene expression, thus precluding the identification of negative regulators of DNA methylation. So far, the only known negative factors in DNA methylation, ROS1 and ROS3 (a protein required for ROS1-mediated demethylation), were isolated from genetic screens using the *RD29A::LUC* transgene system [8,9]. Therefore, *RD29A::LUC* happens to be a target of ROS1-mediated demethylation. As mentioned above, the relatively lower number of ROS1/DML2/DML3 target loci in the genome as compared to the number of RdDM loci suggests the presence of unknown negative factors for methylation acting independently of, or in combination with, active demethylation by ROS1/DML2/DML3. Consequently, it is valuable to develop additional RdDM reporter transgenes inserted into different genomic locations to allow for the identification of these negative players.

Here, we report the establishment of a firefly *LUCIFERASE* (*LUC*)-based reporter transgene driven by a dual *35S* promoter that harbors DNA methylation in CG, CHG, and CHH contexts in *Arabidopsis*. We show that *LUC* expression is repressed mainly through CHH methylation in an RdDM-dependent manner. *MOM1* also plays a role in DNA methylation and TGS of the reporter. More importantly, the moderate level of basal *LUC* expression in wild-type plants allows for genetic screens that aim at the isolation of mutants with not only defective but also enhanced DNA methylation. In fact, a *ros1* allele with reduced transgene expression was isolated using this system. The reporter line will prove to be an effective tool in dissecting the mechanisms that regulate DNA methylation.

## Results and discussion

### Generation of the luciferase reporter line, *LUCH*

**Figure 1:**
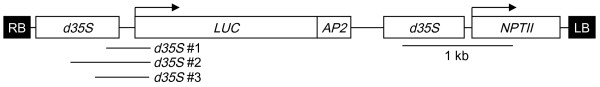
**Structure of *LUCH *and its neighboring transgene.** RB and LB, right border and left border of the T-DNA, respectively. The arrows indicate the directions of the coding regions. The *d35S* fragments (marked #1 to #3) specific for the *d35S* promoter upstream of *LUC* are amplified by PCR following digestion with the restriction enzyme McrBC as well as in bisulfite sequencing.

Initially, we aimed to establish a *LUC*-based transgene that reported both TGS by RdDM and post-transcriptional gene silencing by miRNAs to allow for forward genetic screens. A transgene was constructed such that *LUC* was C-terminally fused in frame to the partial *AP2* fragment containing the miR172 binding site [10] and the transgene was driven by a dual *35S* promoter, which will be referred to as *d35S*, from *Cauliflower Mosaic Virus* (*d35S::LUC-AP2*). In the same vector, *d35S*-driven *NEOMYCIN PHOSPHOTRANSFERASE II* (*d35S::NPTII*) served as a selectable marker for plant transformation (Figure 1). This construct was introduced into the *rna-dependent rna polymerase6–11* (*rdr6–11*) [11-13] mutant background to prevent sense transgene post-transcriptional silencing (S-PTGS; [11-13]) and one line with moderate levels of LUC signal was isolated to enable bidirectional genetic screens based on higher or lower LUC signals. The *d35S::LUC-AP2* transgene in this line was named *LUCH* (*LUC* repressed by CHH methylation), as we found later that it was repressed by CHH methylation in *d35S*. *LUCH* was a one-copy insertion at a single genomic locus according to Southern blot analysis using the *LUC* sequence as a probe (Additional file 1: Figure S1). Thermal asymmetric interlaced PCR (TAIL-PCR) followed by sequencing revealed that the transgene resided 20 nucleotides before the stop codon of At3g07350, a gene of unknown function. This insertion did not cause any obvious morphological phenotypes.

### *LUCH* does not report miRNA activity

Since *LUCH* contained a miR172 binding site, we first investigated whether it was able to report miRNA activity. If it were repressed by miR172, we would expect mutations in miRNA biosynthesis genes (reviewed in [14]), such as *DICERLIKE1* (*DCL1*), *HYPONASTIC LEAVES1* (*HYL1*), and *SERRATE* (*SE*) to de-repress *LUCH* expression. In the F2 population of *LUCH* crossed to *dcl1–7*, LUC luminescence was moderately increased in 12 out of 216 segregating seedlings (Additional file 1: Figure S2A). Since *LUCH* and *DCL1* are not linked, the small proportion of seedlings with the moderately high LUC luminescence was not consistent with *dcl1–7* being able to de-repress *LUCH* expression. Indeed, genotyping confirmed that only one of the 12 was homozygous for *dcl1–7*, and three of the 12 were homozygous for the wild-type *DCL1* allele. Therefore, the moderate increase was likely due to inherent variations in *LUCH* expression or other background mutations. *hyl1* and *se-1* mutations also failed to increase LUC luminescence [see Additional file 1: Figure S2B and S2C. These results demonstrate that *LUCH* was unable to report miRNA activities even though the *LUC* transcript contains a miRNA-binding site in the 3′ UTR.

### *LUCH* is regulated by RdDM-mediated TGS

**Figure 2:**
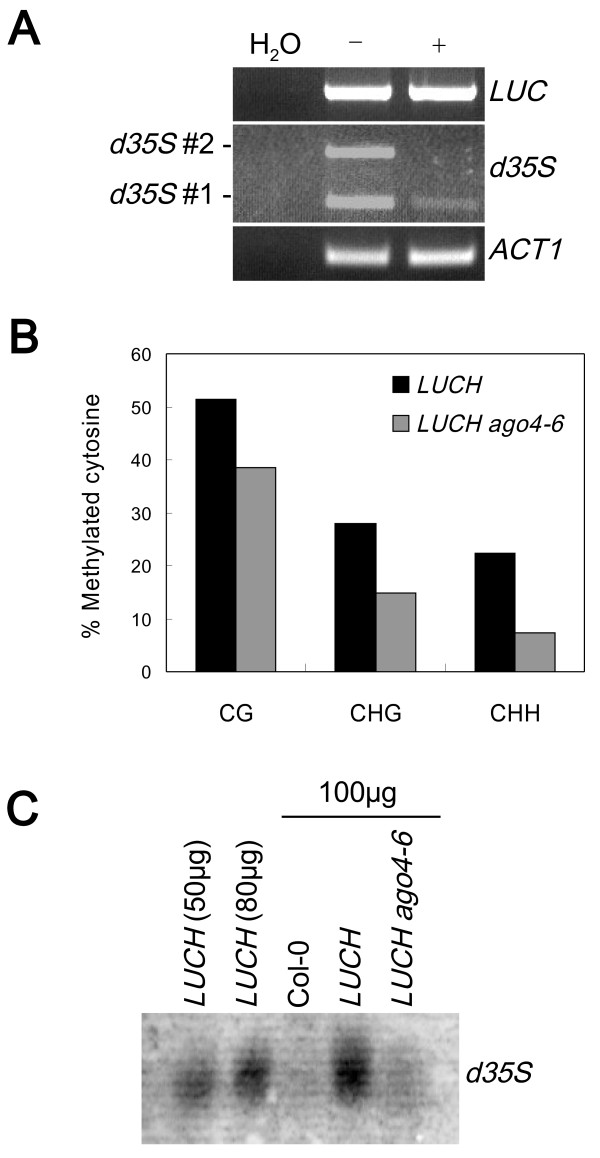
**Molecular characteristics of *LUCH *associated with RdDM. (A)** Analysis of DNA methylation in the *d35S* and the *LUC* coding region in *LUCH* by McrBC-PCR. The two *d35S* fragments are as diagrammed in Figure 1. − and + indicate McrBC-untreated and treated genomic DNA, respectively. ‘H_2_O’ is a negative control PCR without genomic DNA. McrBC digests methylated DNA to result in reduced PCR product amounts. **(B)** Bisulfite sequencing analysis of cytosine methylation in *d35S* in *LUCH* in wild type and *ago4–6*. The top strand of *d35S* #3 in Figure 1 was analyzed. **(C)***d35S*-specific siRNA accumulation in the *LUCH* line as detected by northern blotting. The numbers indicate the amount of enriched small RNAs loaded into the gel. Col-0, wild type (with no transgene).

To evaluate whether *LUCH* was repressed by RdDM-mediated TGS, we first examined whether *LUCH* had the molecular characteristics associated with RdDM. When compared with other reporter systems (*NOSpro* and *α’pro*[15,16]), *d35S* is more than twice as long as those promoters but has a similar percentage of GC content. *d35S* has a relatively high non-CG composition (23 CG, 19 CHG and 138/128 CHH in forward/reverse strands), which was also observed in the *α’pro* system that was reported to be more sensitive to the regulation by RdDM than *NOSpro*[15,16]. McrBC-PCR was conducted using primers that specifically amplified the *d35S* in *LUCH* instead of that in *d35S::NPTII* to evaluate the DNA methylation status of the *LUCH* transgene. The results showed that *d35S* was methylated whereas the *LUC* coding region was not (Figure 2A). Bisulfite sequencing revealed the presence of DNA methylation in CG, CHG, and CHH contexts (Figure 2B). The levels of CHH methylation were 22%, which was particularly high compared to other previously established reporter lines of RdDM. For example, the *clk-sk* line had 15% CHH methylation in the *SUPERMAN* 5′ region [17]; the *RD29A::LUC* line had 1% and 6% CHH methylation in the *RD29A* promoter in wild type and *ros1*, respectively [8]. Treatment of *LUCH* seedlings with 5-aza-2′-deoxycytidine, an inhibitor of cytosine methylation increased LUC luminescence and *LUC* transcript levels, indicating that cytosine methylation transcriptionally silenced *LUCH* expression [see Additional file 1: Figure S3.

Next, since RdDM target loci produce siRNAs, we determined the accumulation of siRNAs from the *LUCH* and *d35S::NPTII* transgenes. Even though we did not artificially introduce any hairpin source of *d35S*-specific siRNAs, siRNAs were detected in the *LUCH* line by northern blotting using a *d35S*-specific probe (Figure 2C). High throughput sequencing was conducted to examine the small RNAs from the transgenes in more detail. siRNAs mapping to both DNA strands of the two transgenes were found; and 22 nucleotide siRNAs were the most abundant small RNA species [see Additional file 1: Figure S4A and S4B]. Even though *LUCH* was introduced into *rdr6–11* to prevent S-PTGS by blocking the biogenesis of secondary siRNAs, 21 nucleotide and 22 nucleotide siRNAs mapping to the transgene were present, which suggests that PTGS was still occurring. Perhaps the siRNAs were primary siRNAs resulting from sense and antisense transcription from the locus or secondary siRNAs from the activities of *RDR2*. Twenty four nucleotide siRNAs, which are associated with RdDM, were also present. Among 18 to 27 nucleotide small RNAs that mapped to *d35S* in *LUCH*, 24 nucleotide siRNAs accounted for approximately 19% of the total [see Additional file 1: Figure S4A]. The *d35S* promoters driving *LUC* and *NPTII* were 96% identical in sequences. We took advantage of the sequence differences to determine whether both regions generated siRNAs. Indeed, siRNAs specific to each *d35S* were found [see Additional file 1: Figure S4C; Additional file 2], indicating that each *d35S* gave rise to siRNAs. The reverse strand 24 nucleotide siRNAs were similar in quantity between the two transgenes (123 and 106 reads for *LUCH* and *d35S::NPTII*, respectively). Interestingly, forward strand 24 nucleotide siRNAs were different in quantity between the two transgenes: 509 and 120 reads were from *d35S::NPTII* and *LUCH*, respectively. The abundance of *d35S::NPTII*-specific siRNAs was attributed to both higher diversity of siRNA species and higher levels of a subset of species [see Additional file 2]. The basis for the differential siRNA levels is unknown but may be due to differences in read-through transcription at the two *d35S*. Taken together, *LUCH* exhibits the molecular characteristics associated with RdDM, such as CHH methylation and 24 nucleotide siRNA production.

**Figure 3:**
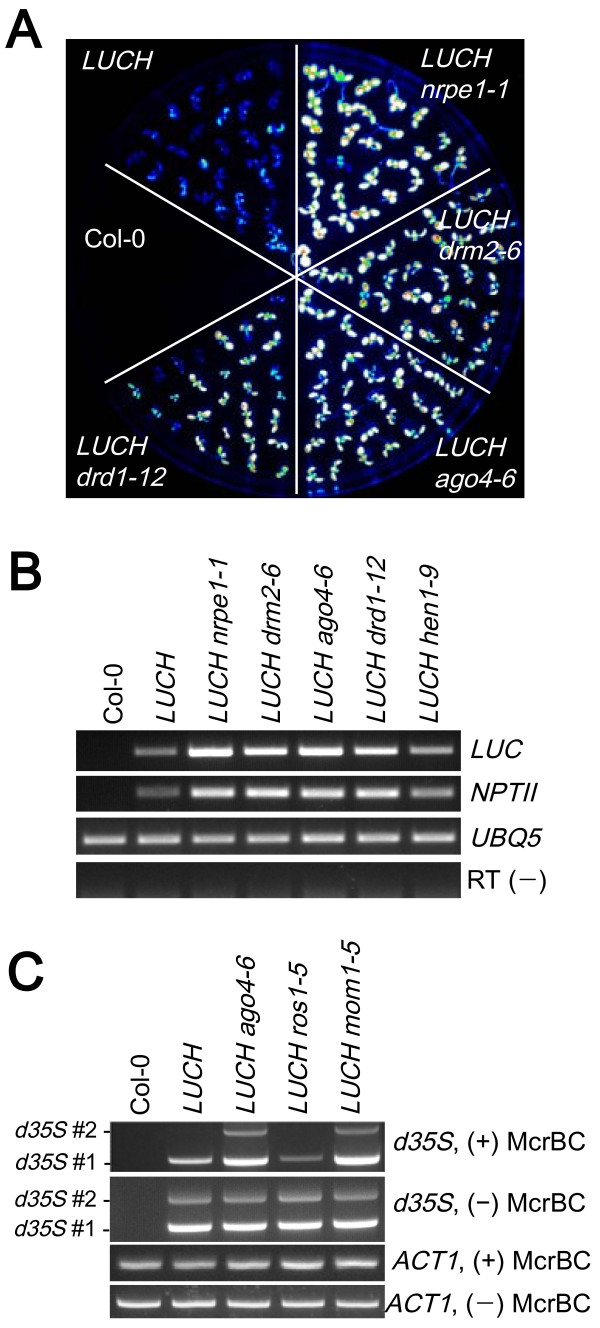
**The RdDM pathway is genetically required for the suppression of *LUCH *expression. (A)** De-repression of LUC luminescence in various RdDM mutants. Each spot represents an *Arabidopsis* seedling. The brighter the spots, the higher the LUC luminescence. Col-0, wild type (with no transgene). **(B)** RT-PCR of *LUC* and *NPTII* in various RdDM mutants. *UBQ5* serves as a loading control. RT (−), *UBQ5* RT-PCR in which the reverse transcription was conducted in the absence of the reverse transcriptase. **(C)** Analysis of cytosine methylation in the *d35S* in *LUCH* in *ago4–6*, *ros1–5* and *mom1–5* mutants by McrBC-PCR. *ACT1* serves as an internal, unmethylated control.

The regulation of *LUCH* by RdDM was further supported by the fact that mutations in known RdDM pathway components de-repressed *LUCH* expression. We mutagenized the *LUCH* line with either ethyl methanesulfonate (EMS) or T-DNA and searched for mutants with higher LUC luminescence (Figure 3A). Genetic analyses demonstrated that each mutant with high LUC luminescence harbored a single, recessive mutation. Map-based cloning revealed that the mutations were in *HUA ENHANCER1*, *AGO4*, *DRM2* and *DEFECTIVE IN RNA-DIRECTED DNA METHYLATION1* (*DRD1*) [see Additional file 1: Figure S5, which are known genes in the RdDM pathway (reviewed in [1]). In addition, introducing *nrpe1–1*, a mutant of the largest subunit of Pol V (reviewed in [2]), into *LUCH* de-repressed LUC luminescence (Figure 3A). These mutants had higher levels of *LUC* transcripts as revealed by RT-PCR (Figure 3B), indicating that the de-repression of *LUCH* expression was at the transcriptional level. Since both *LUC* and *NPTII* are under the regulation of *d35S*, we analyzed the expression levels of *NPTII* by RT-PCR. The *NPTII* transcript levels were also increased in these RdDM mutants (Figure 3B). We next analyzed the DNA methylation status of *d35S* in these mutants. Southern blot analysis with a *d35S*-specific probe showed that *d35S*-specific bands were downwardly shifted in *ago4–6*, *drd1–12* and *drm2–6* [see Additional file 1: Figure S6, indicating that DNA methylation at *d35S* was reduced in *ago4–6, drd1–12* and *drm2–6*. Bisulfite-sequencing with primers that allowed only amplification of the *d35S* in *LUCH* showed that the levels of DNA methylation were decreased in all sequence contexts in *ago4–6*, with CHH methylation being the most drastically decreased (Figure 2B). These results show that *LUCH* is repressed by *de novo* DNA methylation at *d35S* and the repression requires RdDM components. To evaluate whether maintenance methylation at CG and CHG contexts by *MET1* and *CMT3*, respectively, contributes to the repression of *LUCH*, we crossed *met1–3* and *cmt3–7* mutations into *LUCH*. *met1–3* or *cmt3–7* did not affect *LUCH* expression [see Additional file 1: Figure S7, indicating that this reporter line was mainly repressed by *de novo* methylation through *DRM2*. These molecular and genetic results demonstrate that *LUCH* faithfully reports RdDM-mediated TGS.

### *LUCH* is regulated by *MOM1*

**Figure 4:**
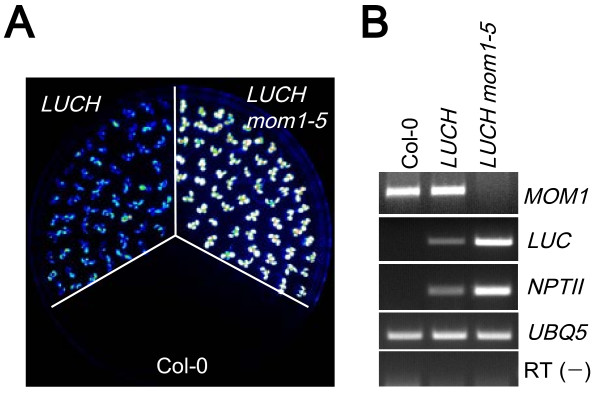
***LUCH *is regulated by MOM1. (A)** De-repression of LUC luminescence in *LUCH mom1–5*. **(B)** RT-PCR of *LUC*, *NPTII* and *MOM1* in wild type (Col-0), *LUCH* and *LUCH mom1–5.*

Our genetic screen also resulted in the isolation of a new *mom1* allele (*mom1–5*) that displayed de-repressed LUC luminescence (Figure 4A; [see Additional file 1: Figure S5]). RT-PCR confirmed the increased levels of *LUC* and *NPTII* transcripts and the absence of *MOM1* transcripts in the mutant (Figure 4B). DNA methylation at *d35S* was moderately decreased in *mom1–5*, as revealed by McrBC-PCR and Southern blot analysis (Figure 3C; [see Additional file 1: Figure S6]). The reduction in DNA methylation in *mom1–5* was less severe than in RdDM mutants [see Additional file 1: Figure S6]. Nonetheless, this shows that the DNA methylation and TGS of *LUCH* require *MOM1*.

### *LUCH* is regulated by *ROS1*-mediated DNA demethylation

**Figure 5:**
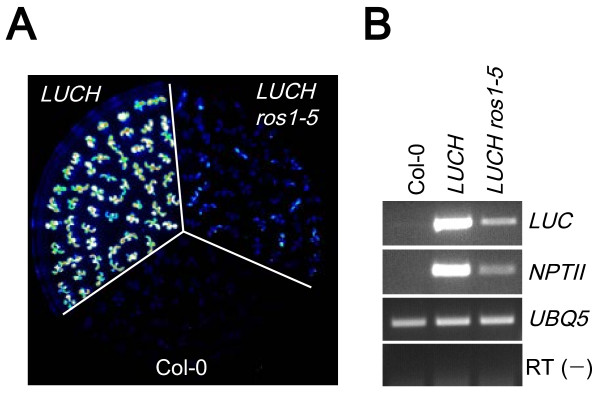
***LUCH *is targeted by *ROS1*-mediated DNA demethylation. (A)** Reduction of LUC luminescence in *LUCH ros1–5*. Note that the images were taken with the same exposure conditions as in Figures 3A and 4A, but *LUCH* appeared much brighter here because the contrast was adjusted to better reflect the differences between *LUCH* and *LUCH ros1–5*. **(B)** RT-PCR of *LUC* and *NPTII* in wild type (Col-0), *LUCH* and *LUCH ros1–5.*

A major motivation to establish a *LUC*-based reporter was to enable the screening for mutants with enhanced silencing. The *LUCH* line, which exhibited a moderate basal level of LUC luminescence, was suitable for such a purpose. We performed T-DNA insertional mutagenesis of the *LUCH* line and isolated a recessive mutant allele with lower levels of LUC luminescence (Figure 5A). Map-based cloning identified this mutant as a new allele of *ROS1* [see Additional file 1: Figure S5], a gene required for DNA demethylation. This suggested that loss of demethylation resulted in the accumulation of cytosine methylation in *d35S* and reinforcement of TGS of *LUCH*. Indeed, there was an increase in DNA methylation of *d35S* in *LUCH* in *ros1–5* according to McrBC-PCR (Figure 3C). Levels of *LUC* and *NPTII* transcripts were decreased as determined by RT-PCR (Figure 5B). In addition, treatment of *LUCH ros1–5* seedlings with 5-aza-2′-deoxycytidine increased the expression of *LUCH* to wild-type levels [see Additional file 1: Figure S3], which further supported the notion that increased DNA methylation in *ros1–5* led to enhanced TGS of *LUCH*. Therefore, even though *LUCH* is transcriptionally repressed by RdDM, the basal expression of *LUCH* is relatively high such that the transgene can be used to screen for mutants with enhanced silencing.

## Conclusions

We developed a transgenic *LUC* reporter system that reports both TGS by RdDM and *MOM1*, and *ROS1*-mediated demethylation. Moderate expression of the reporter enables genetic screens in two directions to isolate mutants with decreased as well as increased DNA methylation. Considering that existing TGS reporter systems, such as the *NOSpro*, *α’pro*, and *clk-sk* lines, are mainly suitable for the isolation of positive players in RdDM, *LUCH* is a useful genetic resource for the identification of negative players in RdDM, for which nothing is known. Moreover, *LUCH* will potentially contribute to the better understanding of *MOM1*-mediated TGS or the mechanisms of active demethylation. For the latter, although *RD29::LUC* reports *ROS1*-mediated DNA demethylation, as a second reporter of *ROS1*-mediated demethylation residing at a different genomic location, *LUCH* will enrich our resources to tackle the mechanisms of demethylation.

## Methods

### Plant material

*Arabidopsis* mutants used in this study were *rdr6–11 *[11], *dcl1–7 *[18], *se-1 *[19], *hyl1 *[20], *met1–3 *[21], *cmt3–7 *[17] and *drd3–1 *[22] and newly isolated *drm2–6*, *ago4–6*, *drd1–12*, *hen1–9*, *ros1–5* and *mom1–5*. For map-based cloning of newly isolated mutants, *LUCH rdr6–11* in the Columbia-0 (Col-0) accession was introgressed into Landsberg *erecta* (L*er*) by backcrossing to L*er* five times and one line with a similar level of LUC activity as *LUCH* in Col-0 was isolated. The isolated mutants from *LUCH rdr6–11* in Col were each crossed to *LUCH rdr6–11* in L*er*, and in the F2 population, seedlings with high (for *drm2–6*, *ago4–6*, *drd1–12*, *hen1–9*, and *mom1–5)* or low (*ros1–5*) luciferase activities were identified and served as the mapping population. Polymorphisms between Col-0 and L*er* were utilized to map and clone the genes.

### Growth conditions and luciferase live imaging

*Arabidopsis thaliana* seeds were surface-sterilized, planted on MS-agar plates containing 1% sucrose, and stratified at 4 °C for three days. Seedlings were grown at 23 °C under continuous lights for ten days. All experiments were performed with ten-day old seedlings unless otherwise specified. For luciferase live imaging, 1 mM luciferin (a substrate of luciferase; Promega, Madison, Wisconsin, USA) in 0.01% Triton X-100 was sprayed onto the seedlings, which were then transferred to a Stanford Photonics Onyx Luminescence Dark Box. Luciferase images were taken with a Roper Pixis 1024B camera controlled by the WinView32 software at a two minute exposure time. Identical exposure conditions were used to capture all images in this study. The images were displayed and analyzed with WinView32 such that image contrast was adjusted to effectively distinguish the difference in intensities between different lines within a plate as previously described [23].

### Construction of transgene, southern blot analysis and TAIL-PCR

The *LUC* coding region was amplified using the Rlucp1 and Rlucp2 primers and pRL-SV40 (Promega) as the template. *d35S::LUC* was constructed by replacing *GFP* in pAVA321 [24] with the *LUC* coding region using *Nco*I and *BamH*I restriction sites. The *d35S::LUC* cassette was cloned into the pPZP211 [25] at the *Sal*I and *BamH*I restriction sites. An *AP2* fragment including the miR172 binding site was amplified from Col-0 genomic DNA with the primers AP2p26 and AP2p28 and inserted downstream of *d35S::LUC* in pPZP211 using *BamH*I and *EcoR*I to generate *d35S::LUC-AP2*, which will be referred to as *LUCH*. The construct was introduced into *rdr6–11* plants by *Agrobacterium tumefaciens*-mediated transformation. Southern blot analysis was performed according to the standard protocol [26] to evaluate the copy number of *LUCH* using the full-length *LUC* coding region as the probe. The probe was amplified with the primers lucp6 and lucp7, and radiolabeled with the RPN1633 Rediprime II Random Prime Labeling System (GE Healthcare Biosciences, Pittsburgh, Pennsylvania, USA). TAIL-PCR was performed as described [27]. Primers used are listed in Additional file 3: Table S1.

### Analysis of DNA cytosine methylation

For the McrBC-PCR assay, two reactions were set up for each genomic DNA sample: McrBC-treated and untreated reactions. A total of 300 ng genomic DNA was digested with 3 units of McrBC (New England Biolabs, Ipswich, Massachusetts, USA) for 25 minutes at 37 °C in a 20 μl reaction. Using 1 μl (15 ng) of restricted genomic DNA as the template, genomic regions corresponding to *d35S* or full length *LUC* in the *LUCH* transgene were amplified using the 35Sf and LUC 0.13 k R primers or the lucp6 and lucp7 primers, respectively. *ACT1* was amplified with the Actin1-F and Actin1-R primers and used as a loading control. PCR products were analyzed on a 2% agarose gel stained with ethidium bromide. For Southern blot analysis, 15 μg of genomic DNA was digested with *Alu*I (NEB) and hybridization was performed following standard methods [28]. The *d35S* promoter was PCR-amplified with the 35Sf and 35Sr primers and radiolabeled using the RPN1633 Rediprime II random prime labeling system (GE Healthcare). For bisulfite sequencing, 1 μg of genomic DNA was subjected to bisulfite conversion using the EpiTect Bisulfite Kit according to the manufacturer’s instructions (Qiagen, Hilden, Germany). Converted DNA was subjected to PCR reactions with primers YZ 35 S Bis F and YZ LUC Bis R and the PCR products were cloned into the pGEM-T Easy vector (Promega). At least 26 colonies were sequenced for each sample. Unique clones were obtained and analyzed for DNA methylation with Kithmeth (http://katahdin.mssm.edu/kismeth/revpage.pl) [28]. For 5-aza-2′-deoxycytidine (Sigma, St. Louis, Missouri, USA) treatment, seeds were germinated and grown on MS-agar medium containing 7 μg/ml of the chemical for two weeks and luciferase images were taken. Primers used are listed in Additional file 3: Table S1.

### Analysis of small RNA accumulation

RNA isolation and hybridization to detect small RNAs were performed as described previously [29]. To detect siRNAs from the *d35S* promoter, a DNA fragment was amplified from the *d35S* promoter using the 35Sf and 35Sr primers and cloned into the pGEM-T Easy vector (Promega). The plasmid was linearized by *Spe*I (NEB) and used as a template for *in vitro* transcription with T7 RNA polymerase (Promega) in the presence of [α-^32^P] UTP. The labeled *in vitro* transcripts were used as the probe in northern blotting. Radioactive signals were detected with a Phosphorimager. For small RNA deep sequencing, a small RNA library was constructed using the TruSeq Small RNA Sample Prep Kit (Illumina, San Diego, California, USA) according to the manufacturer’s instructions with some modifications. Instead of total RNA, 15 to 40 nucleotide long RNAs were used as the starting material. The small RNA library was sequenced by Illumina Hiseq2000 at the genomics core facility at the University of California Riverside. After the raw reads were filtered by the Illumina quality control pipeline and the adaptor sequences were trimmed, 14,363,865 reads between 18 nucleotides and 28 nucleotides were matched to the *Arabidopsis* genome (TAIRv10) as well as the transgenes with SOAP2 [30]. A total of 8,710,699 and 22,245 reads were mapped to the *Arabidopsis* genome and the transgenes, respectively, with no mismatches.

### RT-PCR

cDNA was synthesized from 5 μg of DNaseI (Roche, Basel, Switzerland)-treated total RNA using Reverse Transcriptase (Fermentas, Burlington, Ontario, Canada) and oligo-dT (Fermentas) as the primer. Using cDNA and gene-specific primers, PCR was performed and RT-PCR products were analyzed on a 2% agarose gel stained with ethidium bromide. The sequences of primers are listed in Additional file 3: Table S1.

## Abbreviations

d35S: dual 35 S promoter from Cauliflower mosaic virus; LUC: Luciferase; *LUCH*: *LUC* repressed by CHH methylation; RdDM: RNA-directed DNA methylation; RT-PCR: reverse transcriptase-polymerase chain reaction; siRNA: small interfering RNA; TAIL-PCR: thermal asymmetric interlaced PCR; TGS: transcriptional gene silencing; UTR: untranslated region.

## Competing interests

The authors declare that they have no competing interests.

## Authors’ contributions

SYW and XC wrote the manuscript. SYW did the RT-PCR, McrBC-PCR, 5aza-dC treatments, the genetic analysis of *LUCH*, and identified two mutants to be *drm2–6* and *ros1–5* through map-based cloning. SL performed the siRNA northern blot, Southern blot analysis and TAIL-PCR and mapped the *drd1–12* mutant. BZ transformed the reporter construct and selected the *LUCH* line. YZ mapped *ago4–6*. XZ mapped *mom1–5* and performed Southern blot analysis. DL mapped *hen1–9* and constructed the small RNA library. HY did McrBC-PCR. LG analyzed the small RNA library data. TTD did bisulfite sequencing. XC constructed the reporter plasmid, conceived and guided the project. All authors read and approved the final manuscript.

## Supplementary Material

Additional file 1 **Figure S1.** Southern blot analysis determines the *LUCH* transgene copy number. (A) A map of *LUCH* and its neighboring transgene. The positions of the *EcoR*I and *Hind*III restriction sites, the expected sizes of restriction fragments and the position of the *LUC* probe are shown. (B) Southern blot analysis of *LUCH*. Genomic DNA from Col (wild type) or the *LUCH* line was digested with *EcoR*I or *Hind*III and hybridized with a radiolabeled full-length *LUC* probe. The radiolabeled DNA molecular weight standards are shown on the right. The sizes and numbers of bands are consistent with a single copy of *LUCH* at a single genomic location. **Figure S2.***LUCH* is not regulated by the miRNA pathway. (A) LUC images of Col-0, *LUCH*, *LUCH ago4–6* (a positive control showing de-repression of LUC luminescence) and seedlings from the F2 population of *dcl1–7* crossed to *LUCH*. In the F2 population, LUC luminescence was moderately increased in 12 out of 216 segregating seedlings (only six are indicated by circles here). (B) LUC images of Col-0, *LUCH*, *LUCH ago4–6* and *LUCH hyl1*. The *hyl1* mutation did not result in de-repression of LUC luminescence. (C) LUC images of Col-0, *LUCH* and seedlings from the F3 population of *se-1* crossed to *LUCH*. The F2 plant was genotyped to be homozygous for *LUCH* and *rdr6–11* and heterozygous for *se-1*. Therefore, one quarter of the F3 progenies are theoretically homozygous for *se-1*. There was no apparent de-repression of *LUCH* by *se-1*. **Figure S3**. De-repression of *LUCH* and *LUCH ros1–5* by the methylation inhibitor 5-aza-2′-deoxycytidine (5Aza-dC). (A) Seedlings were grown on MS media for ten days (mock) or on 7 μg/ml 5Aza-dC-supplemented MS media for two weeks (5Aza-dC) followed by LUC luminescence imaging. (B) RT-PCR analysis of *LUC* and *NPTII* expression in mock- or 5Aza-dC-treated *LUCH* and *LUCH ros1–5* seedlings. *UBIQUITIN5* (*UBQ5*) was used as a loading control. The RT (−) reactions were performed with *UBQ5* primers. **Figure S4.** Transgene-specific small RNAs in the *LUCH* line as determined by deep sequencing. (A) Size distribution of small RNAs mapping to the entire T-DNA containing *LUCH* and *d35S::NPTII* (total), the *d35S* promoter in *LUCH* (*d35S*) or the *LUC* coding sequence (*LUC*). (B) Distribution and abundance of 24 nucleotide small RNAs mapping to the *LUCH* and *d35S::NPTII* transgenes. Top and bottom figures indicate the distribution of 24 nucleotide siRNAs from forward and reverse strands, respectively. (C) Distribution and abundance of 24 nucleotide small RNAs that are specific to each *d35S* promoter in the two transgenes. The 4% sequence variations between the *d35S* in the two transgenes allowed the identification of these transgene-specific *d35S* siRNAs. Small RNAs mapping to both strands were detected. **Figure S5.** Schematic diagrams of the gene structures and the mutations in the newly isolated mutant alleles in this study. White and black rectangles indicate untranslated regions and coding exons, respectively. Lines represent introns. Arrows in *mom1–5* indicate the primers used for RT-PCR. The new *hen1* allele is not diagrammed because the exact nature of the mutation is not known. The allele was shown by a genetic complementation test with known *hen1* mutants to be a new *hen1* allele. **Figure S6.** Southern blot analysis of cytosine methylation in *d35S*. (A) Map of the transgenes. Bars represent the probe, which should hybridize to both transgene promoters. (B) Genomic DNA was isolated from Col-0, *LUCH*, *LUCH ago4–6* and *LUCH drd1–12*, digested with cytosine methylation-sensitive *Alu*I and hybridized with the radiolabeled *d35S* probe. DNA bands are shifted downward in *ago4–6* and *drd1–12*, indicating that DNA methylation in *d35S* is decreased in *ago4–6* and *drd1–12*. Though the juxtaposed lanes are discontinuous, they are from a single gel. The phosphor-image was taken from a single membrane. (C) Southern blot analysis of Col-0, *LUCH*, *LUCH ago4–6*, *LUCH mom1–5* and *LUCH drm2–6*. DNA bands are shifted downward to a lesser extent in *mom1–5* than in *ago4–6* or *drm2–6*. **Figure S7.***LUCH* is not repressed by *MET1* or *CMT3*. (A) LUC imaging of seedlings from an F3 population of *cmt3–7* crossed to *LUCH*. The F2 plant was genotyped to be homozygous for *LUCH* and *rdr6–11* and heterozygous for *cmt3–7*. Therefore, one quarter of the F3 progenies are theoretically homozygous for *cmt3–7*. If CMT3 represses *LUCH*, de-repression of *LUCH* is expected in one quarter of the seedlings. No such de-repression was observed. (B) LUC imaging of an F2 population of *met1–3* crossed to *LUCH*. Seedlings with weakly de-repressed LUC signal were identified (circled), genotyped, and found not to be homozygous for *met1–3*. Note that *MET1* and *LUCH* are not linked, such that 3/16 of the seedlings are expected to be *LUCH* (or *LUCH*/*+*) *met1–3*. Click here for file

Additional file 2 Sequences of 24 nucleotide small RNAs mapping specifically to each *d35S* in the two transgenes. Small RNAs were classified based on their transgene origin and strandedness. The sequences of the small RNAs, the number of reads and the positions of their 5′ nucleotides along the construct as shown in Additional file 1: Figure S4 is shown. Click here for file

Additional file 3 **Table S1** DNA oligonucleotides used in this study. Click here for file

## References

[B1] Law JA Jacobsen SE Establishing, maintaining and modifying DNA methylation patterns in plants and animals Nat Rev Genet 2010 11 204 220 10.1038/nrg2719 20142834 PMC3034103

[B2] Haag JR Pikaard CS Multisubunit RNA polymerases IV and V: purveyors of non-coding RNA for plant gene silencing Nat Rev Mol Cell Biol 2011 12 483 492 10.1038/nrm3152 21779025

[B3] Furner IJ Matzke M Methylation and demethylation of the Arabidopsis genome Curr Opin Plant Biol 2011 14 137 141 10.1016/j.pbi.2010.11.004 21159546

[B4] Lister R O’Malley RC Tonti-Filippini J Gregory BD Berry CC Millar AH Ecker JR Highly integrated single-base resolution maps of the epigenome in Arabidopsis Cell 2008 133 523 536 10.1016/j.cell.2008.03.029 18423832 PMC2723732

[B5] Yokthongwattana C Bucher E Caikovski M Vaillant I Nicolet J Mittelsten Scheid O Paszkowski J MOM1 and Pol-IV/V interactions regulate the intensity and specificity of transcriptional gene silencing EMBO J 2010 29 340 351 10.1038/emboj.2009.328 19910926 PMC2824458

[B6] Vaillant I Schubert I Tourmente S Mathieu O MOM1 mediates DNA-methylation-independent silencing of repetitive sequences in Arabidopsis EMBO Rep 2006 7 1273 1278 10.1038/sj.embor.7400791 17082821 PMC1794702

[B7] Amedeo P Habu Y Afsar K Mittelsten Scheid O Paszkowski J Disruption of the plant gene MOM releases transcriptional silencing of methylated genes Nature 2000 405 203 206 10.1038/35012108 10821279

[B8] Zheng X Pontes O Zhu J Miki D Zhang F Li WX Iida K Kapoor A Pikaard CS Zhu JK ROS3 is an RNA-binding protein required for DNA demethylation in Arabidopsis Nature 2008 455 1259 1262 10.1038/nature07305 18815596 PMC2782394

[B9] Gong Z Morales-Ruiz T Ariza RR Roldan-Arjona T David L Zhu JK ROS1, a repressor of transcriptional gene silencing in Arabidopsis, encodes a DNA glycosylase/lyase Cell 2002 111 803 814 10.1016/S0092-8674(02)01133-9 12526807

[B10] Chen X A microRNA as a translational repressor of APETALA2 in Arabidopsis flower development Science 2004 303 2022 2025 10.1126/science.1088060 12893888 PMC5127708

[B11] Peragine A Yoshikawa M Wu G Albrecht HL Poethig RS SGS3 and SGS2/SDE1/RDR6 are required for juvenile development and the production of trans-acting siRNAs in Arabidopsis Genes Dev 2004 18 2368 2379 10.1101/gad.1231804 15466488 PMC522987

[B12] Mourrain P Beclin C Elmayan T Feuerbach F Godon C Morel JB Jouette D Lacombe AM Nikic S Picault N Rémoué K Sanial M Vo TA Vaucheret H Arabidopsis SGS2 and SGS3 genes are required for posttranscriptional gene silencing and natural virus resistance Cell 2000 101 533 542 10.1016/S0092-8674(00)80863-6 10850495

[B13] Dalmay T Hamilton A Rudd S Angell S Baulcombe DC An RNA-dependent RNA polymerase gene in Arabidopsis is required for posttranscriptional gene silencing mediated by a transgene but not by a virus Cell 2000 101 543 553 10.1016/S0092-8674(00)80864-8 10850496

[B14] Chen X Small RNAs and their roles in plant development Annu Rev Cell Dev Biol 2009 25 21 44 10.1146/annurev.cellbio.042308.113417 19575669 PMC5135726

[B15] Matzke M Aufsatz W Kanno T Daxinger L Papp I Mette MF Matzke AJ Genetic analysis of RNA-mediated transcriptional gene silencing Biochim Biophys Acta 2004 1677 129 141 10.1016/j.bbaexp.2003.10.015 15020054

[B16] Aufsatz W Mette MF Matzke AJ Matzke M The role of MET1 in RNA-directed de novo and maintenance methylation of CG dinucleotides Plant Mol Biol 2004 54 793 804 10.1007/s11103-004-0179-1 15604652

[B17] Lindroth AM Cao X Jackson JP Zilberman D McCallum CM Henikoff S Jacobsen SE Requirement of CHROMOMETHYLASE3 for maintenance of CpXpG methylation Science 2001 292 2077 2080 10.1126/science.1059745 11349138

[B18] Robinson-Beers K Pruitt RE Gasser CS Ovule development in wild-type Arabidopsis and two female-sterile mutants Plant Cell 1992 4 1237 1249 12297633 10.1105/tpc.4.10.1237 PMC160211

[B19] Prigge MJ Wagner DR The Arabidopsis SERRATE gene encodes a zinc-finger protein required for normal shoot development Plant Cell 2001 13 1263 1280 11402159 10.1105/tpc.13.6.1263 PMC135584

[B20] Lu C Fedoroff N A mutation in the Arabidopsis HYL1 gene encoding a dsRNA binding protein affects responses to abscisic acid, auxin, and cytokinin Plant Cell 2000 12 2351 2366 11148283 10.1105/tpc.12.12.2351 PMC102223

[B21] Saze H Scheid OM Paszkowski J Maintenance of CpG methylation is essential for epigenetic inheritance during plant gametogenesis Nat Genet 2003 34 65 69 10.1038/ng1138 12669067

[B22] Kanno T Huettel B Mette MF Aufsatz W Jaligot E Daxinger L Kreil DP Matzke M Matzke AJM Atypical RNA polymerase subunits required for RNA-directed DNA methylation Nat Genet 2005 37 761 765 10.1038/ng1580 15924141

[B23] Chinnusamy V Stevenson B Lee BH Zhu JK Screening for gene regulation mutants by bioluminescence imaging Sci STKE 2002 2002 pl10 12107339 10.1126/stke.2002.140.pl10

[B24] von Arnim AG Deng XW Stacey MG Cloning vectors for the expression of green fluorescent protein fusion proteins in transgenic plants Gene 1998 221 35 43 10.1016/S0378-1119(98)00433-8 9852947

[B25] Hajdukiewicz P Svab Z Maliga P The small, versatile pPZP family of Agrobacterium binary vectors for plant transformation Plant Mol Biol 1994 25 989 994 10.1007/BF00014672 7919218

[B26] Sambrook J Fritsch EF Maniatis T Molecular Cloning: A Laboratory Manual 1989 2 Cold Spring Harbor, NY: Cold Spring Harbor Laboratory Press

[B27] Liu Y-G Chen Y High-efficiency thermal asymmetric interlaced PCR for amplification of unknown flanking sequences Biotechniques 2007 43 649 656 10.2144/000112601 18072594

[B28] Gruntman E Qi Y Slotkin RK Roeder T Martienssen R Sachidanandam R Kismeth: analyzer of plant methylation states through bisulfite sequencing BMC Bioinforma 2008 9 371 10.1186/1471-2105-9-371 PMC2553349 18786255

[B29] Park W Li J Song R Messing J Chen X CARPEL FACTORY, a dicer homolog, and HEN1, a novel protein, act in microRNA metabolism in Arabidopsis thaliana Curr Biol 2002 12 1484 1495 10.1016/S0960-9822(02)01017-5 12225663 PMC5137372

[B30] Li R Yu C Li Y Lam T-W Yiu S-M Kristiansen K Wang J SOAP2: an improved ultrafast tool for short read alignment Bioinformatics 2009 25 1966 1967 10.1093/bioinformatics/btp336 19497933

