# Supplementary material for: Development of a luciferase-based reporter of transcriptional gene silencing that enables bidirectional mutant screening in Arabidopsis thaliana
Source: Silence. 2012 Jun 7;3:6. doi: 10.1186/1758-907X-3-6 (PMC3548752; doi:10.1186/1758-907X-3-6)
Supplement: Additional file 1 — Figure S1. Southern blot analysis determines the LUCH transgene copy number. (A) A map of LUCH and its neighboring transgene. The positions of the EcoRI and HindIII restriction sites, the expected sizes of restriction fragments and the position of the LUC probe are shown. (B) Southern blot analysis of LUCH. Genomic DNA from Col (wild type) or the LUCH line was digested with EcoRI or HindIII and hybridized with a radiolabeled full-length LUC probe. The radiolabeled DNA molecular weight standards are shown on the right. The sizes and numbers of bands are consistent with a single copy of LUCH at a single genomic location. Figure S2.LUCH is not regulated by the miRNA pathway. (A) LUC images of Col-0, LUCH, LUCH ago4–6 (a positive control showing de-repression of LUC luminescence) and seedlings from the F2 population of dcl1–7 crossed to LUCH. In the F2 population, LUC luminescence was moderately increased in 12 out of 216 segregating seedlings (only six are indicated by circles here). (B) LUC images of Col-0, LUCH, LUCH ago4–6 and LUCH hyl1. The hyl1 mutation did not result in de-repression of LUC luminescence. (C) LUC images of Col-0, LUCH and seedlings from the F3 population of se-1 crossed to LUCH. The F2 plant was genotyped to be homozygous for LUCH and rdr6–11 and heterozygous for se-1. Therefore, one quarter of the F3 progenies are theoretically homozygous for se-1. There was no apparent de-repression of LUCH by se-1. Figure S3. De-repression of LUCH and LUCH ros1–5 by the methylation inhibitor 5-aza-2′-deoxycytidine (5Aza-dC). (A) Seedlings were grown on MS media for ten days (mock) or on 7 μg/ml 5Aza-dC-supplemented MS media for two weeks (5Aza-dC) followed by LUC luminescence imaging. (B) RT-PCR analysis of LUC and NPTII expression in mock- or 5Aza-dC-treated LUCH and LUCH ros1–5 seedlings. UBIQUITIN5 (UBQ5) was used as a loading control. The RT (−) reactions were performed with UBQ5 primers. Figure S4. Transgene-specific small RNAs in the LUCH line as determin [file 1758-907X-3-6-S1.ppt]

## Slide 1
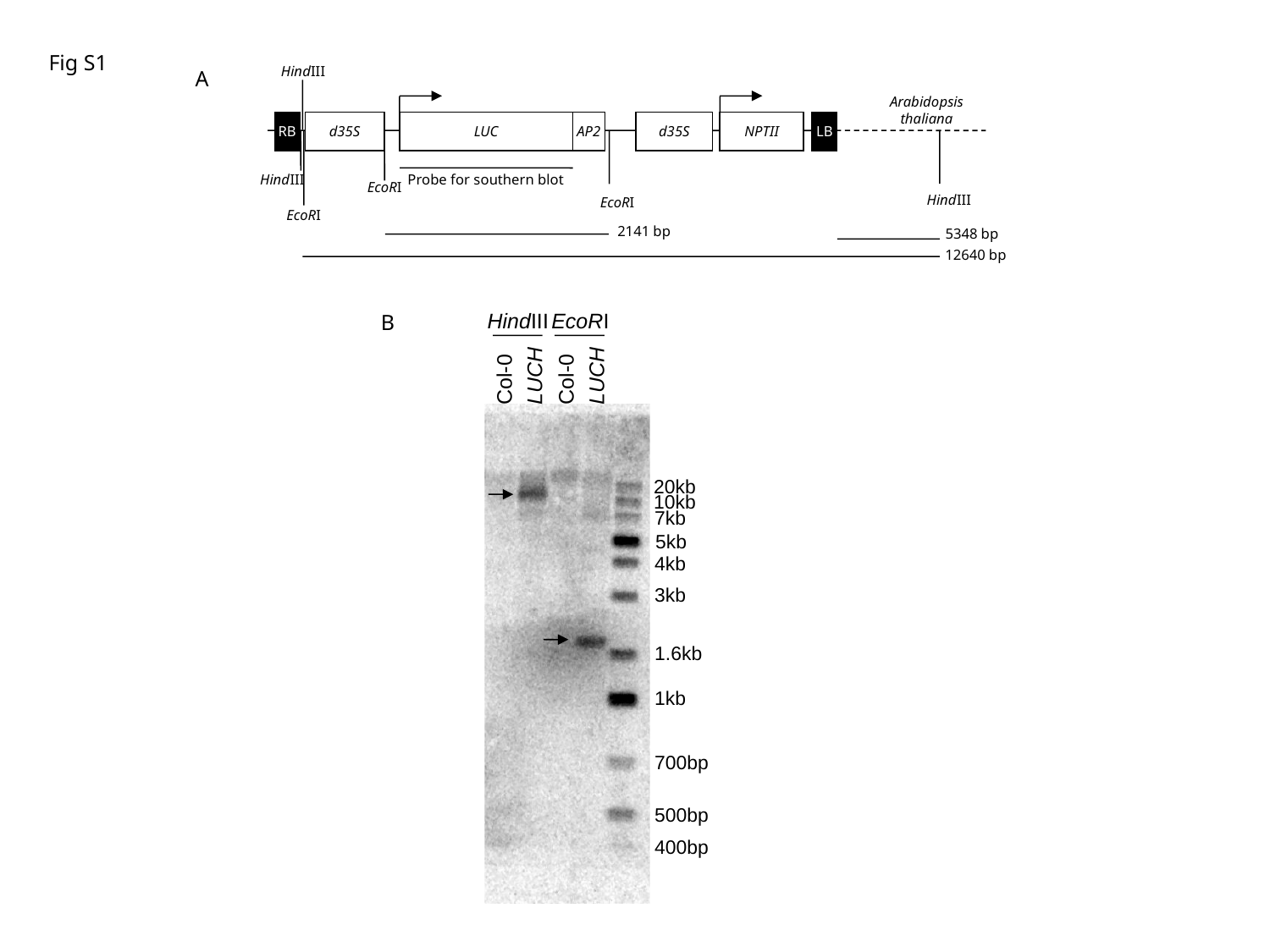

Fig S1
HindIII
Arabidopsis
thaliana
RB
d35S
LUC
AP2
d35S
NPTII
LB
Probe for southern blot
HindIII
EcoRI
HindIII
EcoRI
EcoRI
2141 bp
5348 bp
12640 bp
A
HindIII
Col-0
LUCH
EcoRI
Col-0
LUCH
20kb
10kb
7kb
5kb
4kb
3kb
1.6kb
1kb
700bp
500bp
400bp
B

## Slide 2
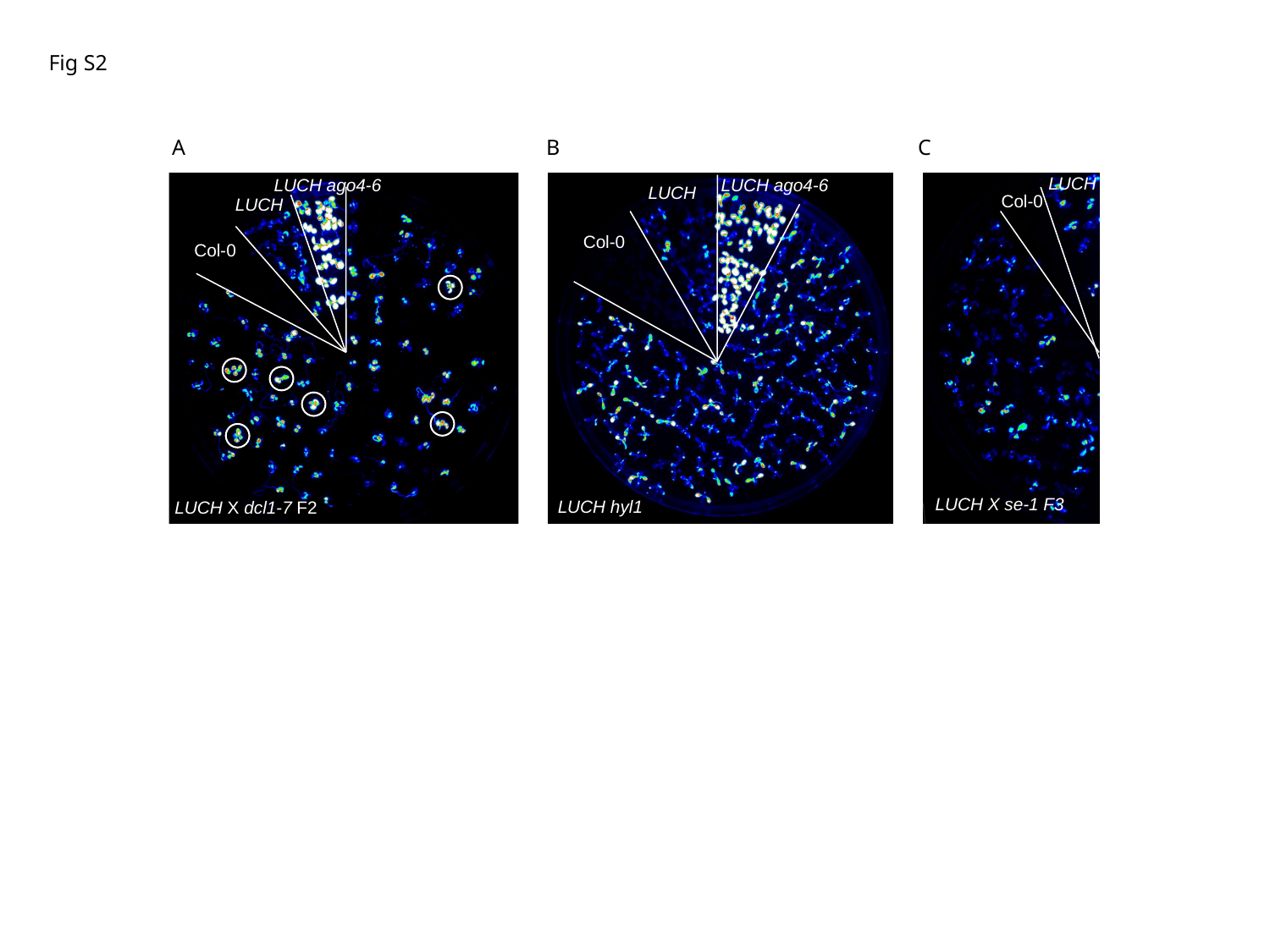

Fig S2
A
B
C
LUCH
LUCH ago4-6
LUCH ago4-6
LUCH
Col-0
LUCH
Col-0
Col-0
LUCH X se-1 F3
LUCH hyl1
LUCH X dcl1-7 F2

## Slide 3
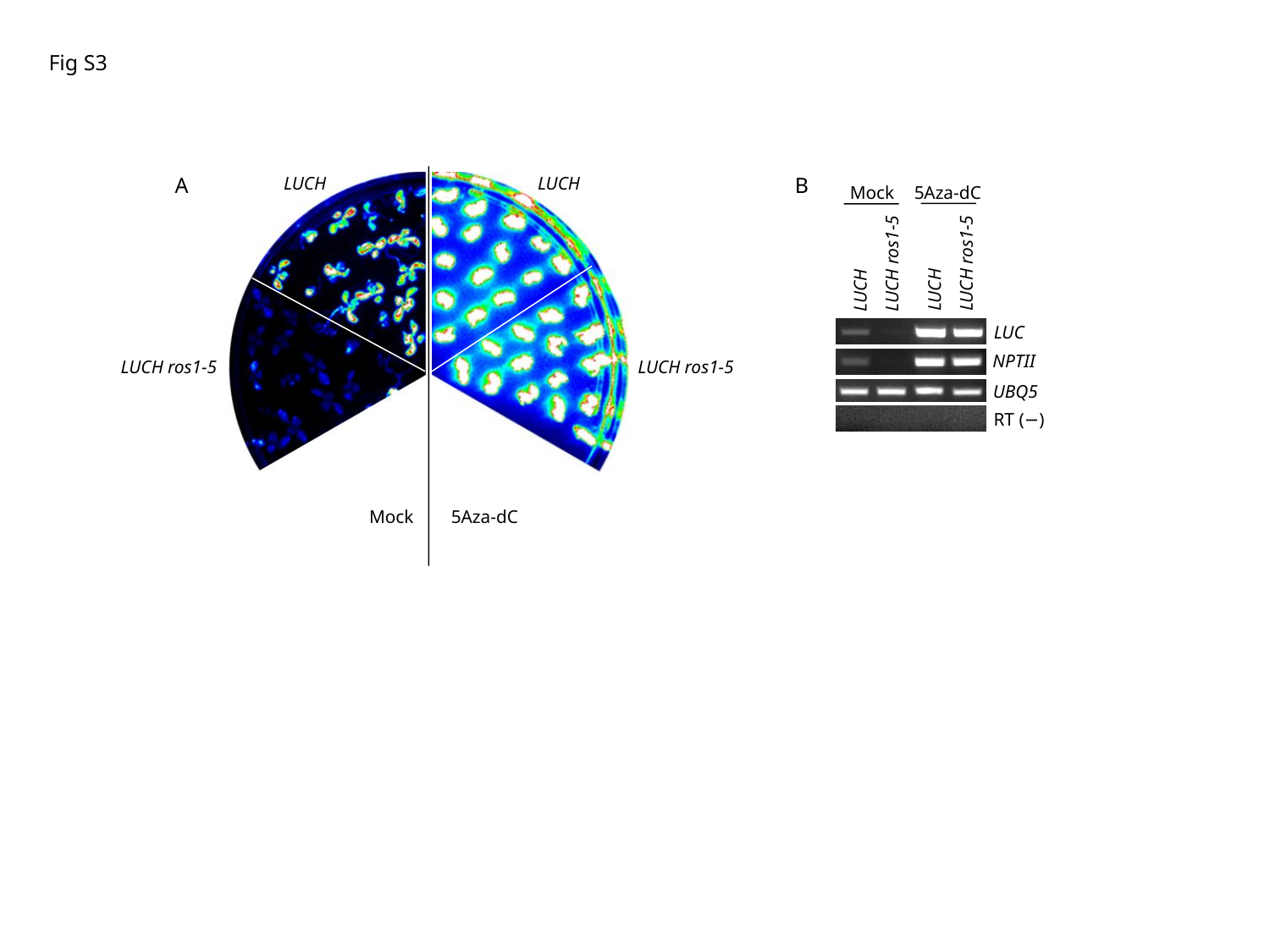

Fig S3
A
LUCH
LUCH
B
5Aza-dC
Mock
LUCH
LUCH ros1-5
LUCH
LUCH ros1-5
LUC
NPTII
UBQ5
RT (−)
LUCH ros1-5
LUCH ros1-5
Mock
5Aza-dC

## Slide 4
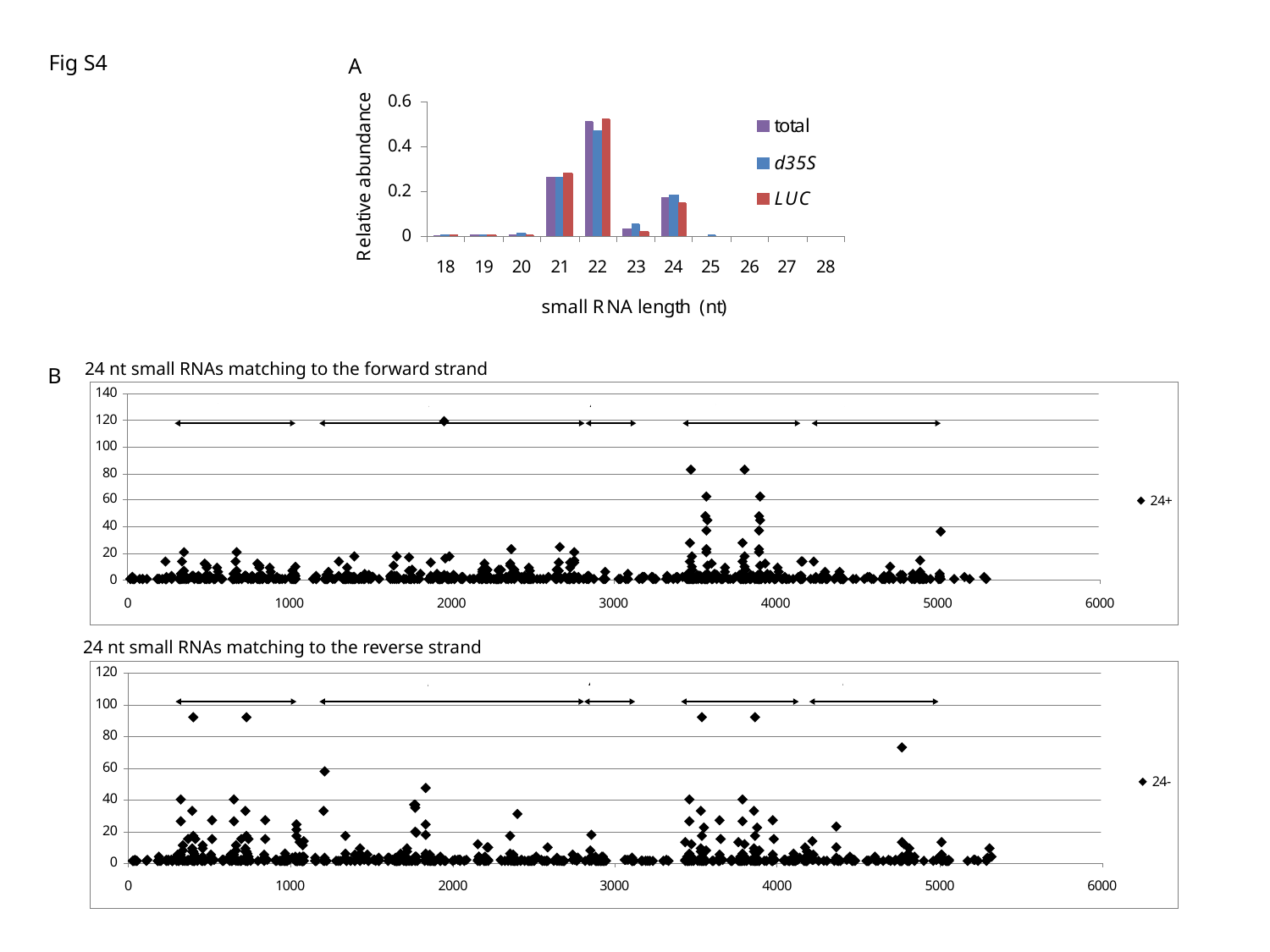

Fig S4
A
24 nt small RNAs matching to the forward strand
B
24 nt small RNAs matching to the reverse strand

## Slide 5
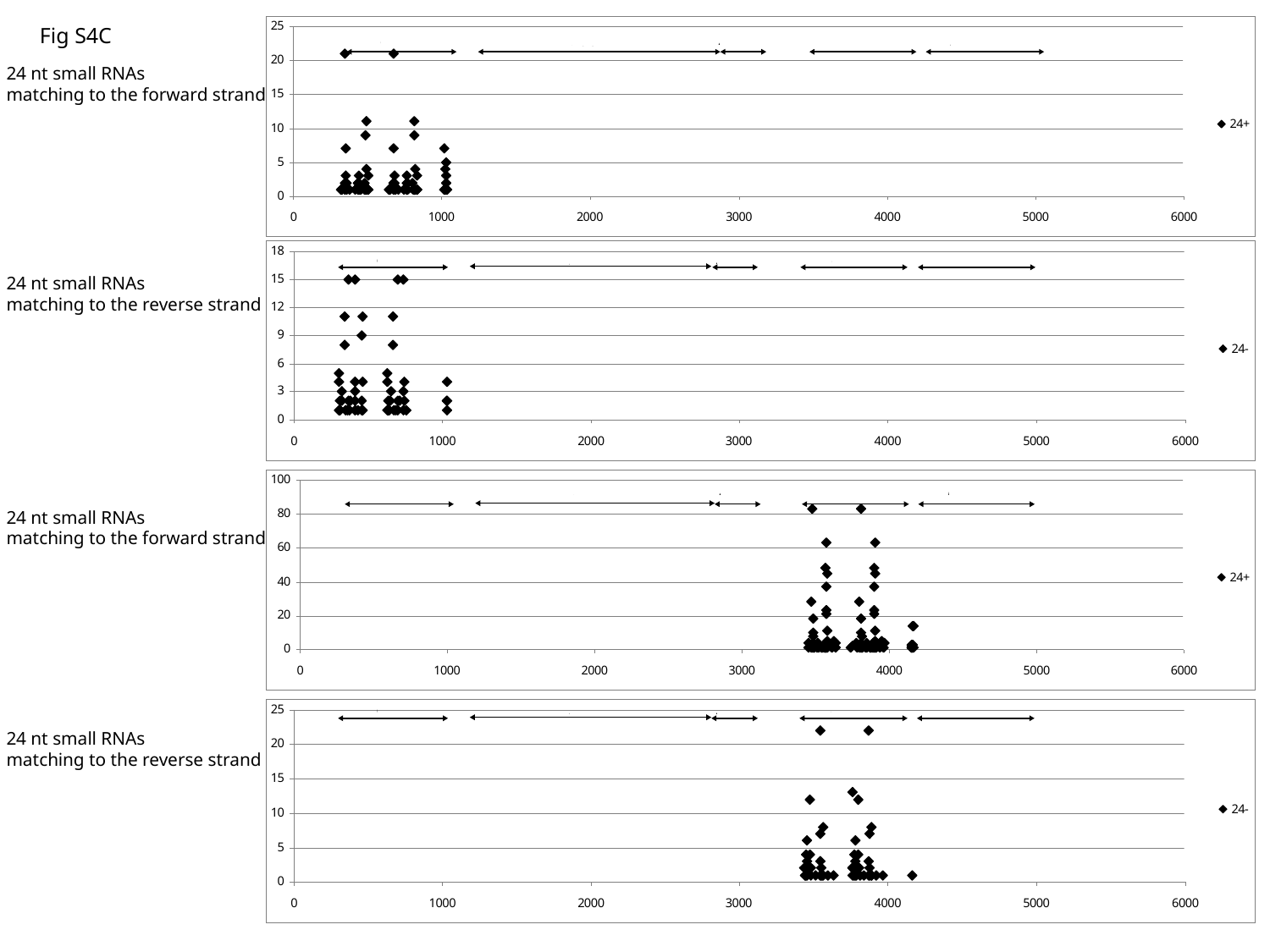

Fig S4C
24 nt small RNAs
matching to the forward strand
24 nt small RNAs
matching to the reverse strand
24 nt small RNAs
matching to the forward strand
24 nt small RNAs
matching to the reverse strand

## Slide 6
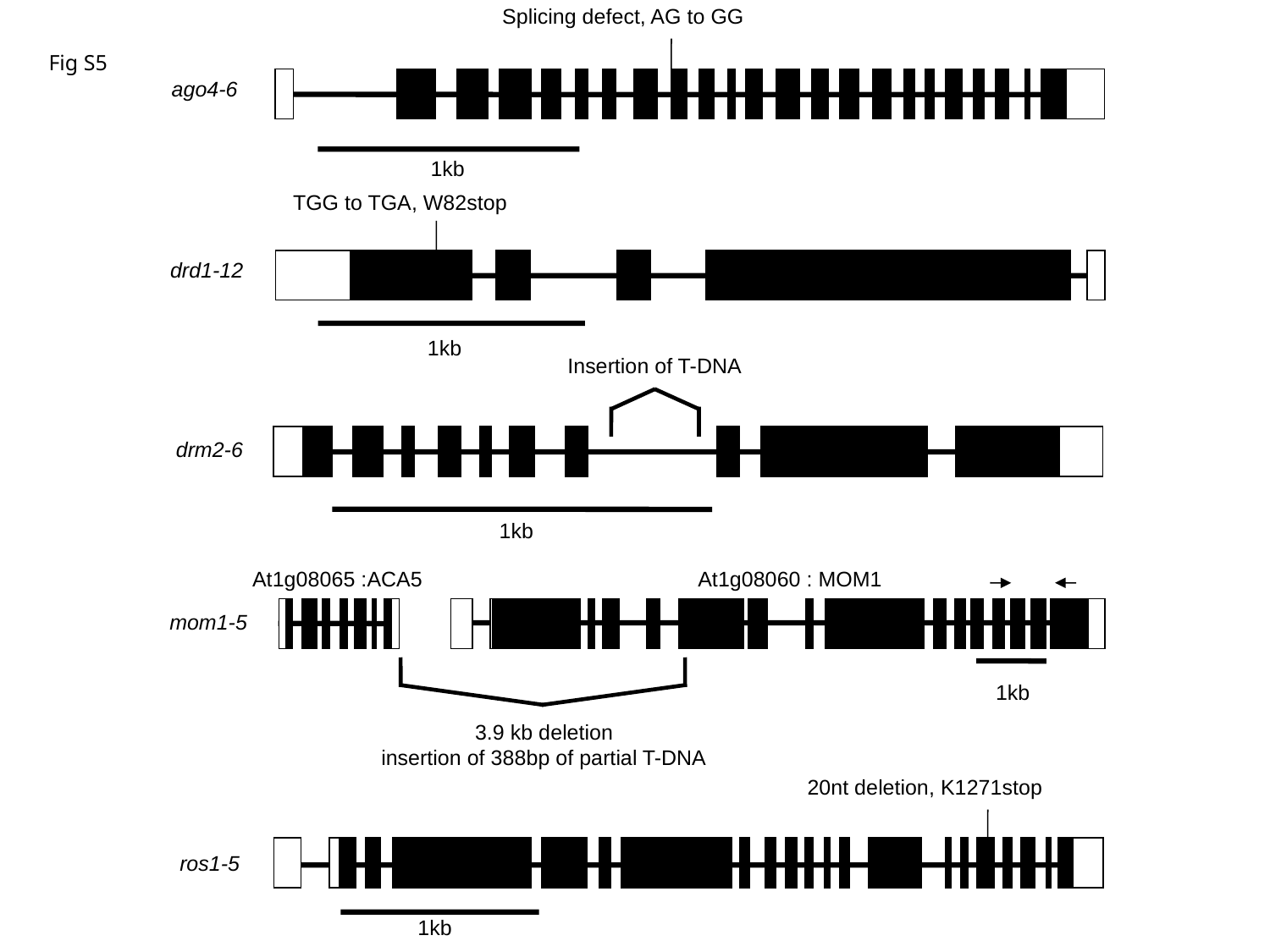

Splicing defect, AG to GG
ago4-6
Fig S5
1kb
TGG to TGA, W82stop
drd1-12
1kb
Insertion of T-DNA
drm2-6
1kb
At1g08065 :ACA5
At1g08060 : MOM1
mom1-5
1kb
3.9 kb deletion
insertion of 388bp of partial T-DNA
20nt deletion, K1271stop
1kb
ros1-5

## Slide 7
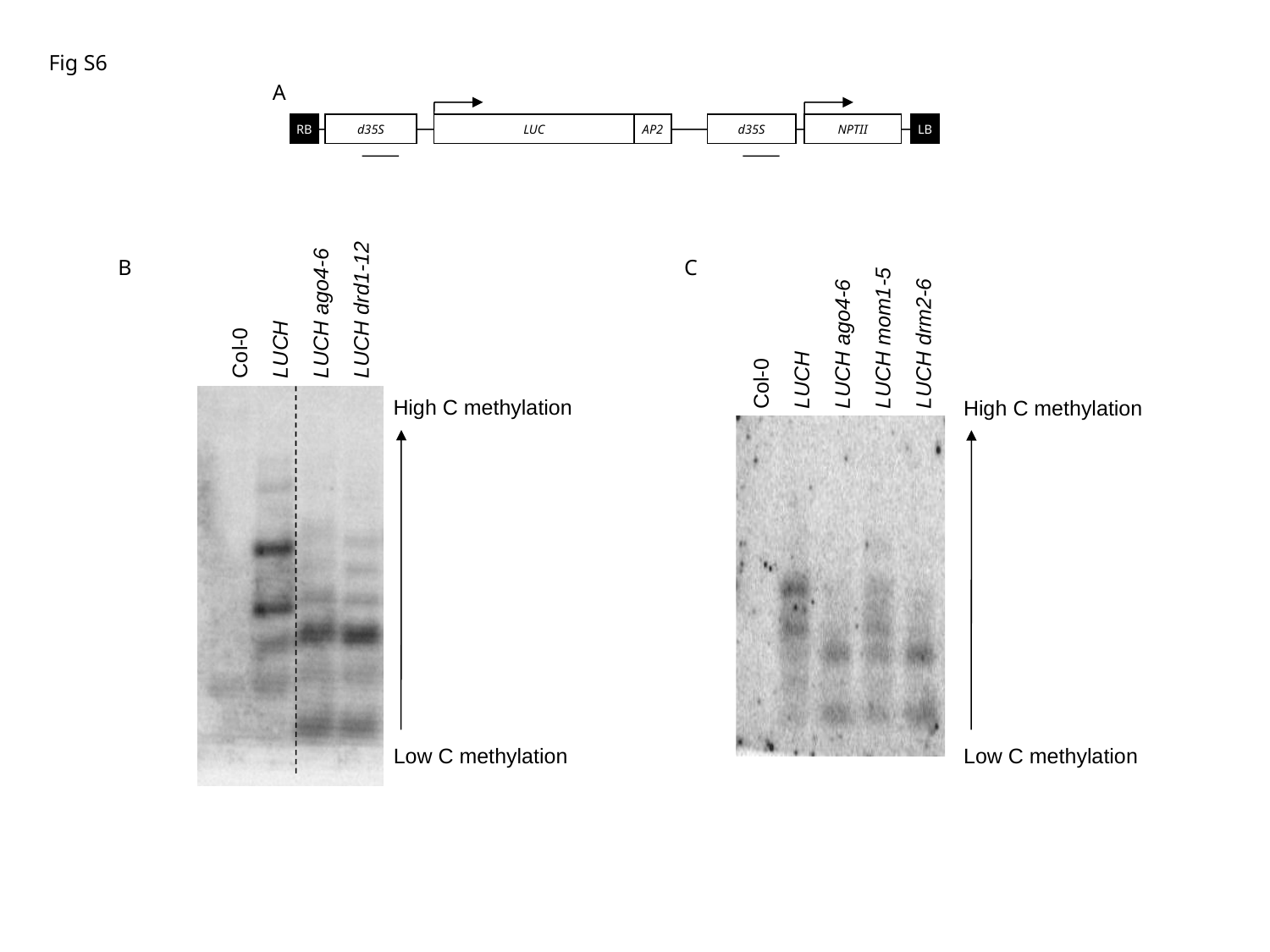

Fig S6
A
RB
d35S
LUC
AP2
d35S
NPTII
LB
Col-0
LUCH
LUCH ago4-6
LUCH drd1-12
Col-0
LUCH
LUCH ago4-6
LUCH mom1-5
LUCH drm2-6
B
C
High C methylation
High C methylation
Low C methylation
Low C methylation

## Slide 8
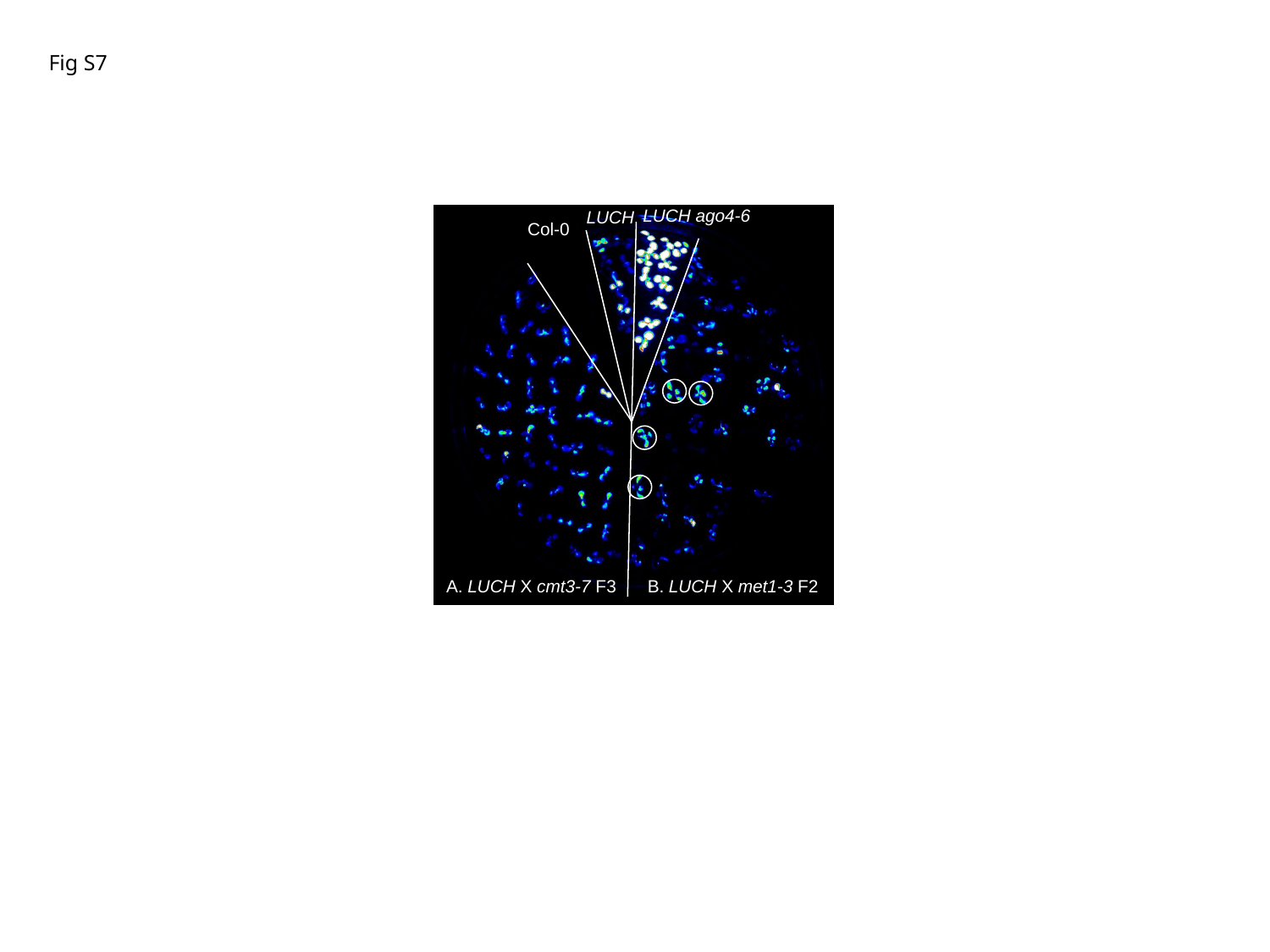

Fig S7
LUCH ago4-6
LUCH
Col-0
A. LUCH X cmt3-7 F3
B. LUCH X met1-3 F2
