# Supplementary material for: Development of a luciferase-based reporter of transcriptional gene silencing that enables bidirectional mutant screening in Arabidopsis thaliana
Source: Silence. 2012 Jun 7;3:6. doi: 10.1186/1758-907X-3-6 (PMC3548752; doi:10.1186/1758-907X-3-6)
Supplement: Additional file 3 — Table S1 DNA oligonucleotides used in this study. [file 1758-907X-3-6-S3.doc]

**Table S1.** DNA oligonucleotides used in this study.

| Name | Sequence | Purpose |
| --- | --- | --- |
| AP2p26 | CCGGTTTGATGGTCGGGCCTCGAC | partial AP2 amplification |
| AP2p28 | GTTTTTTTTAATTACCTTTAGAAAAAGGGA | partial AP2 amplification |
| Rlucp1 | AGGGGATCCACCATGGCTTCGAAAGTTTATGATCCAGAAC | LUC amplification |
| Rlucp2 | TCTAGGATCCTTGTTCATTTTTGAGAACTCGCTC | LUC amplification |
| lucp6 | GCACCCGGGGAAGACGCCAAAAACATAAAAGAAA | McrBC-PCR, southern blot |
| lucp7 | GGACCCGGGTGCGATCTTTCCGCCCTTCTTGGCCT | McrBC-PCR, southern blot |
| Actin1-F | CCAAGCAGCATGAAGATCAA | McrBC-PCR |
| Actin1-R | TGAACAATCGATGGACCTGA | McrBC-PCR |
| 35Sf | CAAAGCAAGTGGATTGATGTGA | McrBC-PCR, southern blot |
| 35Sr | TTTCCACGATGCTCCTCGT | Southern blot |
| LUC 0.13k R | TATGTGCATCTGTAAAAGCAA | McrBC-PCR |
| YZ 35S Bis F | AttAtTGTyGGtAGAGGtATtTTGAAyGATAGtt | Bisulfite sequencing |
| YZ LUC Bis R | CATCTaTAAAAaCAATTaTTCCAaaAACCAaa | Bisulfite sequencing |
| N_UBQ5 | GGTGCTAAGAAGAGGAAGAAT | RT-PCR, loading control |
| C_UBQ5 | CTCCTTCTTTCTGGTAAACGT | RT-PCR, loading control |
| LUCmF5 | CTCCCCTCTCTAAGGAAGTCG | RT-PCR for LUC |
| LUCmR5 | CCAGAATGTAGCCATCCATC | RT-PCR for LUC |
| Kan-RT-F | AGGTTCCATCTGCCAGGTATCA | RT-PCR for NPTII |
| Kan-RT-R | CCCGGTATCCAGATCCACAA | RT-PCR for NPTII |
| At1g08060-F10 | CTCCTATGCCATTATCTTCG | RT-PCR for MOM1 |
| At1g08060-R10 | AACTGATGGAGTTGGAGCTA | RT-PCR for MOM1 |
